# CD38 promotes LPS-induced innate-like activation and proliferation of CD8^+^ T lymphocytes in aged mice

**DOI:** 10.3389/fragi.2025.1701685

**Published:** 2025-12-19

**Authors:** Wendolaine Santiago-Cruz, Enrique Espinosa, Jocelyn C. Pérez-Lara, Héctor Romero-Ramírez, Priyadharshini Devarajan, Fabio García-García, Juan C. Rodríguez-Alba

**Affiliations:** 1 Programa de Doctorado en Ciencias de la Salud, Instituto de Ciencias de la Salud, Universidad Veracruzana, Veracruz, Mexico; 2 Laboratorio de Inmunología Integrativa, Instituto Nacional de Enfermedades Respiratorias (INER), Mexico City, Mexico; 3 Departamento de Biomedicina Molecular, CINVESTAV-IPN, Mexico City, Mexico; 4 Department of Microbiology & Immunology, Renaissance School of Medicine, Stony Brook University, Stony Brook, NY, United States; 5 Departamento de Biomedicina, Laboratorio de Biología del Sueño, Instituto de Ciencias de la Salud, Universidad Veracruzana, Veracruz, Mexico; 6 Departamento de Neuroinmunología, Instituto Nacional de Neurología y Neurocirugía, “Manuel Velasco Suárez”, Mexico City, Mexico

**Keywords:** CD38, lipopolysaccharide, bystander activation, CD8^+^ T cells, aging, inflammaging, T cell subsets

## Abstract

CD38 is a transmembrane glycoprotein involved in NAD^+^ metabolism, calcium signaling, and immune cell activation. Its role in the inflammatory response has been studied extensively in innate immune cells; however, its contribution to the activation of memory T lymphocytes under inflammatory conditions is less understood. Additionally, recent studies have shown an age-related increase in the expression of the protein CD38 in various human and murine tissues. Moreover, CD8^+^ bystander T cells have been shown to contribute to inflammation during the aging process. Given the importance of its potential role in age-related pathologies, we examined the effect of CD38 on bystander activation of CD8^+^ memory T cells in aged mice following lipopolysaccharide challenge. CD38-deficient mice exhibited attenuated serum cytokine responses (IL-1β, IL-6, IFN-γ, and IL-10) and a distinct CD8^+^ T cell profile, characterized by a decrease in activated T cells. Wild-type mice displayed a significant expansion of CD69^+^T_CM_ cells after LPS inoculation, an effect that was absent in CD38-deficient animals. LPS also promoted the expression of CD69 and CD38 in T_EM/EFF_ subsets. Thus, our findings reveal a CD38-dependent mechanism underlying bystander activation of memory CD8^+^ T cells in aging. Highlighting the potential contribution of CD38 to age-related diseases, such as autoimmunity, and in the face of inflammatory conditions in aged people.

## Graphical Abstract

**Figure:**
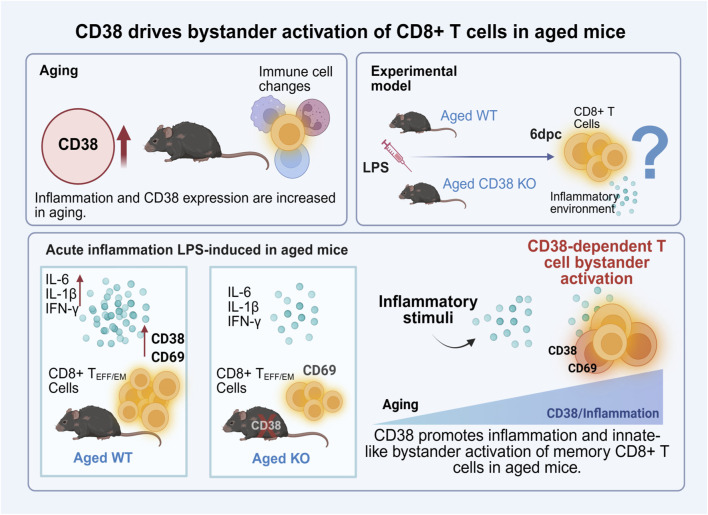

## 1 Introduction

CD38 is a transmembrane glycoprotein expressed on both hematopoietic and non-hematopoietic cells (Zeidler et al., 2022). Its expression was described initially in T lymphocytes (Reinherz et al., 1980). It has a crucial role in Nicotinamide Adenine Dinucleotide (NAD^+^) consumption, cADPR generation, and Ca^2+^ mobilization by its enzymatic activity. It also plays a role in activating effector responses of innate immune cells and T cells during inflammation, influencing the production of cytokines (Amici et al., 2018; Lande et al., 2002; Postigo et al., 2012).

In controlled conditions, inflammation is beneficial for eliminating antigens. It plays a role in regulating the response of various cell subsets, including non-immune cells, innate immune cells, and adaptive immune cells, as well as the activation and differentiation of T lymphocytes (Joshi et al., 2007; Raeber et al., 2018). However, dysregulated immune responses can be detrimental, contributing to a wide range of diseases. The incidence of age-related diseases is rising, and they may be exacerbated by inflammation associated with these diseases, such as autoimmunity, cardiovascular disease, sepsis, and increased susceptibility to viral infections, among others (Carrasco et al., 2022; Ramosa et al., 2017; André et al., 2022; Kim et al., 2018). Additionally, as thymic function declines with age in both mice and humans, the proper response to infections in later stages of life relies on memory cell subpopulations (Xia et al., 2022; den Braber et al., 2012; Tong et al., 2020).

Certain pathological conditions elicit an inflammatory milieu that can trigger antigen-independent immune responses. This phenomenon, known as bystander activation, occurs when T cells become activated independently of TCR engagement. This antigen-independent activation represents an innate-like mode of T cell activation, illustrating how inflammation can shape T cell responses across various pathological conditions (Kim et al., 2018; Le et al., 2023; Taylor et al., 2022; Taylor et al., 2020; Kim and Shin, 2019). In aged mice, TCR-independent activation of T cells during acute inflammation favors the increase of innate-like T cells, IFN-γ production, and confers protection against *Mycobacterium tuberculosis* (Vesosky et al., 2009; Vesosky et al., 2006; Piergallini et al., 2023). Additionally, acute inflammation induced by LPS administration led to changes in T lymphocytes and an increase in CD69^+^ activated T cells (Taylor et al., 2022; Cui et al., 2014; Brinkhoff et al., 2018; Tough et al., 1997).

Recent studies have revealed an age-related rise in the expression of the protein CD38 in diverse human and murine tissues (Camacho-Pereira et al., 2016; Polzonetti et al., 2012). This increased expression, mainly observed in macrophages, is stimulated by the senescence-associated secretory phenotype (SASP) induced by senescent cells (Covarrubias et al., 2020). In chronic infections such as HIV, lipopolysaccharide (LPS) translocation correlates with the expression of CD38 as evidence of an activated T cell phenotype and poor prognosis (Brenchley et al., 2006). CD38 is also stably expressed on CD8+ T cells under inflammatory conditions and is required for CD8+ Tissue-resident (T_RM_) generation (Evrard et al., 2023). Together, these findings suggest that CD38 is associated with aging, elevated levels of inflammatory mediators, and altered states of T cell activation.

Despite the recognized correlation between CD38 and the inflammatory response of innate immune cells, the precise role of CD38 in the effect of inflammation on the functionality and composition of T-lymphocyte subpopulations remains unclear, as well as its consequent effect on T lymphocyte innate-like/bystander activation.

Here, we evaluated CD8^+^ memory T lymphocytes in aged CD38-deficient and wild-type mice after exposure to an acute lipopolysaccharide (LPS) challenge. We utilized the LPS challenge in aged mice to model bystander T cell activation during aging (Taylor et al., 2022; Piergallini et al., 2023). We observed antigen-independent central memory T cell expansion, characterized by the expression of CD69 and Ki-67 in a CD38-dependent manner following LPS challenge. Additionally, elderly mice lacking CD38 were protected from the elevation of IL-1β, IL-6, IFN-γ, and IL-10 levels after LPS administration. These results highlight the significance of CD38 function in the inflammatory response during aging and provide evidence of its role in the innate-like/bystander activation of memory T lymphocytes.

## 2 Materials and methods

### 2.1 Mice

All animal experiments were approved by the institutional Animal Care and Use Committee (2021–01/002). Additionally, the authors followed the guidelines established by official regulation NOM-062-ZOO-1999. Male and female C57BL/6 wild-type (WT) and C57BL/6. CD38^−/−^ (KO) Mice were purchased from Jackson Laboratory (Bar Harbor, ME, United States). Mice were bred by strain to obtain littermates and were housed with food and water available *ad libitum* with a 12-hour light-dark cycle.

Mice were assigned to experimental groups according to availability after applying predefined criteria. Inclusion criteria were 18-month-old WT and CD38 KO mice that had completed the quarantine period. Exclusion criteria included alopecia, physical impairments, or a lack of cohousing for at least 2 weeks prior to the experiments. Animals were removed from the study if they showed signs of illness or compromised welfare. Investigators were blinded during data collection and analysis. Randomization was not applied due to the limited availability of aged mice.

### 2.2 Mice treatment

At the age of 18 months, WT and KO mice were assigned to experimental groups: WT VEH (average weight 30.97 g), WT LPS (32.8 g), KO VEH (29.9 g), and KO LPS (26.7 g). Mice were injected intraperitoneally (i.p.) with sterile saline solution 0.9%, n = 4 per group, or LPS *E. coli* O55:B5 (Lot: 024M4040V, L2880-25 MG, Saint Louis, MO, United States), n = 6 per group at a dose of 0.25 mg/kg body weight once a day for 5 days (Krishna et al., 2016; Chini et al., 2019). For analyses requiring additional samples, the number of animals was increased to 8. 24 hours after the last administration, mice were euthanized by cervical dislocation, and sera and spleens were collected.

Splenocytes were obtained by gently disintegrating spleens through a 200-mesh sterile metal sieve with the plunger of a sterile syringe. Red blood cells were lysed with a 0.85% ammonium chloride solution (NH_4_Cl, 150 mM; KHCO_3_, 10 mM; EDTA, 0.1 mM). Cells were suspended in 1 mL of phosphate-buffered saline (PBS) for viability and cell count determinations. Leucocyte count and viability were determined by trypan blue assay.

For flow cytometry staining panel A: 2 × 10^6^ cells were suspended in PBS and stained with the following fluorophore-conjugated antibodies for extra-cellular staining: anti-CD19-APC-Cy7 (clone: 6D5, Lot: B338086, BioLegend, RRID: AB_830707), anti-CD8-Brilliant Blue-700 (Clone: 53–6.7, Lot: 1048927, BD Horizon, RRID: AB_2744467), anti-CD62L-APC (Clone: MEL-14, Lot: 267555, BD Pharmigen, RRID: AB_398533), anti-CD44-PE-Cy7 (Clone: IM7, Lot: B282420, BD Pharmigen, RRID: AB_1727484), anti-CD38-FITC (Clone: 90, Lot: E004688, eBioscience, RRID: AB_465024), and anti-CD69-PE (Clone: H1.2F3, Lot: B256875, BD Pharmigen, RRID: AB_394508). For the determination of CD4^+^ and CD8^+^ T cell numbers, flow cytometry panel B was used: 2 × 10^6^ cells were suspended in PBS and stained with the following fluorophore-conjugated antibodies: anti CD3-BV605 (Clone:17A2, Lot:0007733, BD Horizon, RRID: AB_2732063), anti CD4-PE (Clone: GK1.5, Lot: B196678, BioLegend, RRID: AB_ 312693), and anti-CD8-Brilliant Blue-700(Clone: 53–6.7, Lot: 1048927, BD Horizon, RRID: AB_2744467). After the addition of the corresponding antibodies, cells were incubated for 15 min at room temperature. After incubation, the cells were washed with PBS and fixed with 1% formaldehyde.

For intracellular staining, the cells were permeabilized with 250 µL of the Cytofix/Cytoperm Fixation/Permeabilization Solution Kit (BD Biosciences, Cat.: 554714, RRID: AB_2869008) for 20 min at 4 °C. Cells were then washed with 1 mL of PermWash 1X. Next, they were stained with anti-Ki67-BV421 (Clone: 21–67, BioLegend, RRID: AB:2562663) overnight at 4 °C. After incubation, the cells were washed with 1 mL of PermWash™ 1X and then fixed with 1% formaldehyde. Cells were analyzed in an LSRFortessa Flow cytometer (BD Biosciences, San Jose, CA, United States) and analyzed using FlowJo V10 Software (BD Biosciences, San Jose, CA, United States). The gating strategy was based on previous reports (Lanzer et al., 2018) and is detailed in Supplementary Figure S2A.

We determined the percentages and absolute numbers of each subpopulation as well as the Mean Fluorescence Intensity (MFI) of activation and proliferation markers. Absolute counts of T lymphocyte subpopulations were calculated by multiplying the percentage of each subpopulation by the total lymphocyte number per spleen sample, as determined by the trypan blue viability count.

$$Absolute\;count=\frac{Subpopulation\;percentage\times Trypan\;blue\;count}{100}$$

### 2.3 Serum cytokine determination

Serum concentrations of interleukin-1 beta (IL-1β), interleukin-6 (IL-6), interferon-gamma (IFN-γ), and interleukin-10 (IL-10) were determined after acute LPS administration using Mouse ELISA^MAX^ DELUXE Set (Biolegend, San Diego, CA) according to the manufacturer’s protocol. For each group (WT or KO), absolute change (Δ) in serum cytokine concentration was calculated as the difference between the mean value in the LPS-treated group and the mean value in the vehicle group (Δ = mean [LPS] − mean [Veh]). The resulting Δ values were used to illustrate the net effect of LPS stimulation, independent of baseline differences between genotypes (Supplementary Figure S1A).

### 2.4 Statistical analysis

Data was tested for normal distribution using the Shapiro-Wilk test, and differences between groups were evaluated using the *t-*test, two-way analysis of variance (ANOVA), or Welch’s ANOVA test to homogenize statistical analysis, followed by the Tukey test or Dunnett T3 as a multiple comparison test, respectively. The levels of significance are represented by asterisks as follows: *p ≤ 0.05, **p ≤ 0.01, ***p ≤ 0.001, and ****p ≤ 0.0001. Data are expressed as mean ± S.D. Data were analyzed with GraphPad Prism 10 software (GraphPad Software, San Diego, CA, United States).

Sample size was limited (n = 4–6 mice per group in flow cytometry panel A; n = 4–8 mice per group in flow cytometry panel B). No formal power calculation was performed prior to the study; group sizes were based on previous similar studies and feasibility considerations. We acknowledge that the statistical power is limited, and results should be interpreted with caution.

### 2.5 Data availability

The raw FCS files and analysis spreadsheets that support the findings of this study are available from the corresponding author upon reasonable request.

## 3 Results

### 3.1 CD38 is essential for cytokine response and CD8^+^ T cell increase after LPS stimulation

**FIGURE 1:**
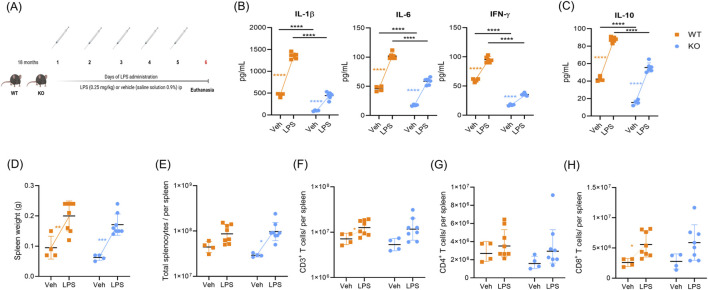
CD38 promotes serum cytokine elevation after acute LPS administration. **(A)** Scheme of acute LPS administration to aged C57BL/6. Cd38^+/+^ (WT) and C57BL/6. Cd38^−/−^ (KO) mice. WT and KO mice were treated with LPS *E. coli* O55:B5 (0.25 mg/kg) or vehicle (veh: saline solution 0.9%) intraperitoneally (i.p.) daily for 5 days (veh n = 4, LPS = 6). Mice were euthanized 24 h after the last LPS challenge. **(B)** Proinflammatory serum cytokines from WT and KO-aged mice. **(C)** IL-10 levels in WT and KO-aged mice. **(D)** Spleen weight of WT and KO aged mice. **(E)** Total splenocytes were counted by trypan blue assay. **(F)** Absolute number of CD3^+^ T cells. **(G)** Absolute numbers of CD3^+^CD4^+^ T cells from aged mice. **(H)** Absolute numbers of CD3^+^CD8^+^ T cells from aged mice. No differences were found between the groups of aged mice. The determination of cytokines was performed in triplicate (Veh, n = 4; LPS, n = 6, independent biological specimens). Flow cytometry panel B was used to determine the counts of CD3^+^, CD4^+^, and CD8^+^ T cells (veh, n = 4, LPS, n = 8, independent biological specimens). The data were analyzed using a two-way ANOVA followed by Tukey’s test. The total CD4^+^ T cell count was analyzed on log10-transformed data to meet normality assumptions; data are plotted on a linear scale. Data are presented as mean ± S.D. *p ≤ 0.05; **p ≤ 0.005; ***p ≤ 0.001; ****p ≤ 0.0001.

To investigate changes in T cell-bystander activation in elderly mice after LPS challenge, and to determine the role of CD38 in these changes, we gave daily intraperitoneal injections of LPS to 18-month-old WT and CD38 KO mice for five consecutive days (Figure 1A). First, we evaluated serum cytokine levels, spleen weight, and the total number of splenocytes. We then assessed the total CD3^+^, CD4^+^, and CD8^+^ cell counts in both WT and KO aged mice in response to LPS administration using flow cytometry panel B.

To evaluate the proinflammatory response, we quantified levels of pro- and anti-inflammatory cytokines in sera from aged WT and KO mice. Under basal conditions, our results show lower levels of cytokines, including IL-1β (*F* (1, 16) = 512.6, *p* < 0.0001), IL-6 (*F* (1, 16) = 260.2, *p* < 0.0001), IFN-γ (*F* (1, 16) = 1,107, *p* < 0.0001), and IL-10 (*F* (1, 16) = 289.7, *p* < 0.0001) in sera from KO mice compared to WT mice (Figures 1B,C). After LPS administration, both WT and KO mice exhibited an increase in serum cytokines compared to baseline conditions; however, CD38-deficient mice had lower levels of all proinflammatory cytokines (Figure 1B; Supplementary Figure S1A). The fold change after LPS challenge was also evaluated, and all the proinflammatory cytokines showed higher fold change compared to baseline levels (Supplementary Figure S1A). The anti-inflammatory cytokine IL-10 followed a similar pattern. In CD38-deficient mice, serum IL-10 increased in the LPS group compared to KO veh (*F* (1, 16) = 610.0, *p* < 0.0001), but it remained lower than in WT LPS-treated mice (Figure 1C). However, the fold change according to baseline levels was not statistically significant (Supplementary Figure S1A).

Aged WT and KO mice showed similar spleen weights and numbers of total splenocytes under basal conditions (*F* (1, 20 = 3.306, *p* = 0.0840), suggesting that CD38 deficiency does not affect spleen weight in aged mice (Figures 1D,E). After LPS administration, both WT and KO mice showed an increase in spleen weight (*F* (1, 20) = 40.6, *p* < 0.0001) (Figure 1D). We also evaluated total CD3^+^, CD4^+^, and CD8^+^ T cell counts; our results showed a higher number of CD3^+^ total T cells (*F* (1, 20) = 7.683, *p* < 0.0118) and CD8^+^ T cells (*F* (1, 20) = 10.31, *p* < 0.0044) in WT aged mice challenged with LPS (Figures 1F–H). No significant differences were found in CD4^+^ T cells (*F* (1, 20) = 0.7459, *p* = 0.3980) (Figure 1G).

### 3.2 CD38 promotes the prevalence of an effector memory T cell phenotype under basal conditions in aged mice

**FIGURE 2:**
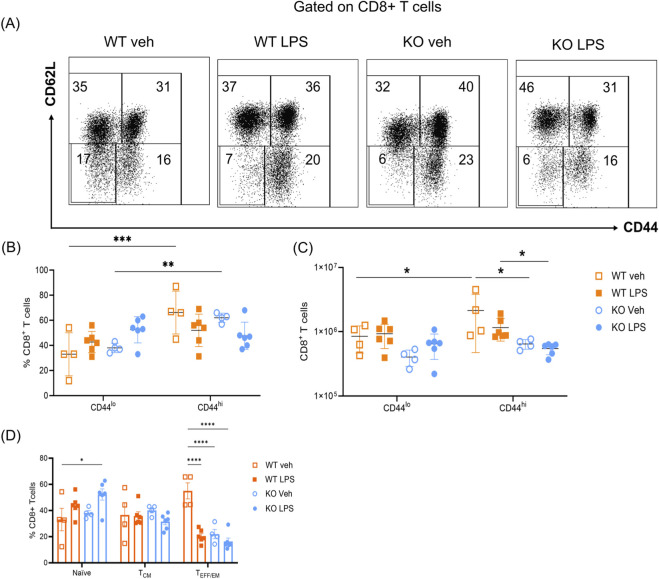
CD38 promotes the enrichment of activated CD44^hi^ CD8^+^ T cells in aged mice. **(A)** Representative dot plots gated on CD19^−^CD8^+^ lymphocytes showing CD44^neg^CD62L^hi^ naïve T cells (naïve), CD44^hi^CD62L^hi^ central memory T cells (T_CM_), CD44^hi^CD62L^low^ effector memory T cells (T_EFF/EM_) CD8^+^ T cells. The numbers in the box represent the average percentage of each T cell subpopulation per group. **(B)** Percentages of total CD44^hi^ (activated T_CM_ and T_EFF/EM_) and CD44^low^ (non-activated) CD8^+^ T cells. **(C)** Absolute numbers of CD44^lo^ and CD44^hi^ CD8^+^ T cells. **(D)** Percentage of naïve, T_CM,_ and T_EFF/EM_ CD8^+^ cells in the four different groups analyzed. Percentages were analyzed using a two-way ANOVA followed by the Tukey test, and absolute numbers were analyzed using log^10^-transformed data to meet the assumptions of normality. Data are plotted on linear scale and presented as mean ± S.D. *p ≤ 0.05; **p ≤ 0.005. The data represent two different experiments (Veh, n = 4; LPS, n = 6, independent biological specimens).

To investigate the influence of CD38 on LPS-induced changes in T lymphocyte subset composition from aged mice, we analyzed the immunophenotype of CD8^+^ T cell subpopulations based on CD44 and CD62L expression to distinguish naïve (CD44^neg^CD62L^hi^), central memory (T_CM_: CD44^hi^CD62L^hi^), and effector/effector memory T cells (T_EFF/EM_: CD44^hi^CD62L^low^) (Figure 2A; Supplementary Figure S2A).

Based on the expression of CD44 on CD8^+^ T cells, we found that in basal conditions, both genotypes showed a higher percentage of CD44^hi^ CD8^+^ T cells (F (1,32) = 17.52, p = 0.0002). This finding is consistent with those in the field that show an increase in antigen-independent T cells in aged mice, known as virtual memory T cells (Renkema et al., 2014). The analysis of CD44^hi^ CD8^+^ T cell numbers supported that CD44^hi^ cells are more abundant in WT aged mice (F (1,32) = 6.637, p = 0.0148). Additionally, in LPS conditions, WT mice exhibited a higher number of CD44^hi^ CD8^+^ T cells compared to KO mice (F (3,32) = 8.135, p = 0.0004). This may reflect that CD38 enhances activation-associated phenotypes in WT mice (Figure 2C). However, when we analyzed CD44 expression after LPS challenge we found that CD38 had no impact on the upregulation of CD44^+^ cells following LPS challenge. The statistical difference between WT LPS and KO LPS CD44^hi^ cells is not a result of the LPS challenge, as no difference was observed compared to baseline conditions (Figure 2C). Our results showed that CD38 did not modify the upregulation of CD44 following LPS stimulation. The difference observed between WT LPS and KO LPS reflects the higher baseline abundance of CD44^hi^ cells in WT mice, rather than an enhanced induction in response to LPS (Figures 2B–D; Supplementary Figure S2B).

### 3.3 CD38 promotes the accumulation of CD8^+^ T cells with an activated phenotype in response to LPS challenge in aged mice

**FIGURE 3:**
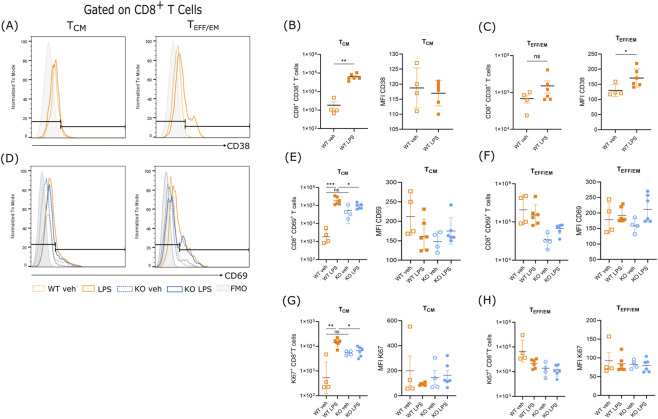
LPS increases the number of memory T cell subpopulations expressing the activation markers CD38 and CD69. **(A)** Representative histograms showing the expression of CD38^+^ in CD8^+^ T_CM_ **(B)** Left: absolute number of CD8^+^CD38^+^ T_CM_ cells. Right: Mean Fluorescence intensity (MFI) of CD38 in CD8^+^ T_CM_ cells. **(C)** Left: absolute number of CD8^+^CD38^+^ T_EFF/EM_ cells. Right: MFI of CD38 in CD8^+^ T_EFF/EM_ cells. MFI was calculated from CD38-positive cells. **(D)** Representative histograms showing the expression of CD69 in CD8^+^ T_CM_ and T_EFF/EM_ cells. **(E)** Left: absolute number of CD8^+^CD69^+^ T_CM_ cells. Right: MFI of CD69 in CD8^+^ T_CM_ cells. **(F)** Left: absolute number of CD8^+^CD69^+^ T_EFF/EM_ cells. Right: MFI of CD69 in CD8^+^ T_EFF/EM_ cells. MFI was calculated from CD69-positive cells. **(G)** Left: Absolute number of CD8^+^Ki67^+^ T_CM_ cells from WT and KO mice. Right: MFI of Ki67 in CD8^+^ T_CM_ cells. **(H)** Left: Absolute number of CD8^+^Ki67^+^ T_EFF/EM_ cells from WT and KO mice. Right MFI of Ki67 in CD8^+^ T_EFF/EM_ cells. Statistical significance for the cell counts of CD38^+^ cells and CD38 MFI was determined using an Unpaired t-test with Welch’s correction. The statistical significance of the CD69 and Ki67 data was determined using a Welch’s ANOVA followed by Dunnett’s T3 as a multiple comparison test. Data are presented as mean ± S.D. Significance levels are indicated as p* ≤0.05; **p ≤ 0.005; ***p ≤ 0.001. The data represent two different experiments (Veh, n = 4; LPS, n = 6, independent biological specimens).

The expression of CD38 is associated with a proinflammatory phenotype in innate cells and metabolic tissues in aged mice, as well as in response to the Senescent Associated Secretory Phenotype (SASP) (Covarrubias et al., 2020; Chini et al., 2019). In addition, the expression of CD38 is not only an activation marker on T cells but could also impact their effector capacities (Chatterjee et al., 2018; Huang et al., 2024). We evaluate CD38 expression on CD8+ T cells after LPS challenge (Figure 3A). First, the CD8^+^CD38^+^T_CM_ absolute number increased in the LPS-challenged group compared to baseline conditions (*t* (5.091) = 6.130, *p* = 0.0015) (Figure 3B, left). However, we did not find differences in the expression of CD38 (*t* (4.608) = 0.4678, *p* = 0.6612) in LPS-challenged mice compared to baseline conditions within this cell phenotype (Figure 3B, right). Additionally, T_EFF/EM_ cells showed an increase in CD38^+^ cells after LPS challenge compared to the WT vehicle group; however, this increase was not statistically significant (*t* (5.918) = 1.456, *p* = 0.1962) (Figure 3C, left). Our data showed an increase in CD38 Median Fluorescence Intensity (MFI) (*t* (7.988) = 2.625, *p* = 0.0305) in LPS-challenged mice (Figure 3C, right).

CD69, a classic marker of T lymphocyte activation, is also expressed by T cells in response to LPS challenge (Le et al., 2023; Vesosky et al., 2006). One study demonstrated a positive correlation between CD38 and CD69 in regulatory T cells; however, there is a lack of evidence on the role of CD38 in the expression of CD69 in memory T cell subsets from aged mice (Taylor et al., 2022). To assess the influence of CD38 on T cell activation in our LPS-challenged mice, we measured CD69 expression in T cell subsets within aged WT and KO mice (Figures 3D–F).

Our data showed that under basal conditions, there were no differences in T_CM_ CD8^+^CD69^+^ T cell numbers between WT and KO mice (*p =* 0.3313). Our results showed higher counts of T_CM_ CD8^+^CD69^+^ cells in aged WT mice after LPS challenge (*p =* 0.0223), and this effect was not observed in CD38-deficient mice (Figure 3E, left). In addition, there was no difference in CD69 density per cell (as measured by CD69-associated fluorescence) between WT and KO mice under basal conditions, and LPS did not alter CD69 expression (*p* = 0.9550) (Figure 3E, right). We observed a tendency for aged WT T_CM_ CD8^+^ cells to express a higher density of CD69 compared to KO mice; however, this difference was not statistically significant (*p* = 0.3382) (Figure 3E, right).

### 3.4 CD38 favors Ki67^+^ CD8^+^ T_CM_ cell expansion in aged mice after LPS administration

To assess the effect of LPS on T cell proliferation, we measured Ki67 expression, a marker of cycling and proliferating cells. This would show the proliferation of antigen-independent bystander T cells, which has been reported in mouse models (Tough et al., 1997).

CD8^+^ T lymphocytes, baseline measurements revealed no significant differences in the number of cells expressing Ki67 in CD8^+^ T cell subpopulations between WT and KO mice (*p* = 0.1012). Additionally, Ki67 baseline MFI did not differ between baseline groups, either in T_CM_ (*p* = 0.9304) or T_EFF/EM_ CD8^+^ T cells (*p* = 0.9426) (Figures 3G,H). Our data showed that LPS administration promoted the expansion of CD8^+^T_CM_ cells expressing Ki67, but only in the presence of CD38 (WT group) (*p* = 0.0037). This effect was not observed in KO mice (Figure 3G). There were no differences in the expression per-cell density of Ki67 (mean fluorescence intensity) in CD8^+^ T cell subpopulations between WT and KO mice, and LPS did not induce any change (Figures 3G,H, right panels).

### 3.5 LPS challenge promotes the increase of a double-activated phenotype, CD69^+^CD38^+^, in T_EM_ CD8^+^ cells in aged mice

**FIGURE 4:**
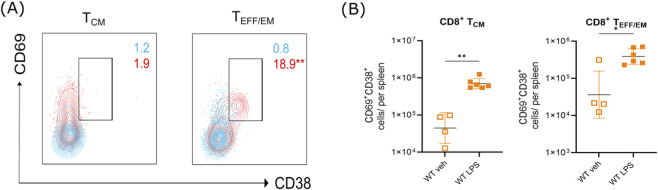
LPS stimulation promotes the increase of CD69^+^CD38^+^ cytotoxic T cells in aged mice. **(A)** Representative contour plots of CD8^+^ T cell subpopulations showing the co-expression of CD69 and CD38 from aged WT mice stimulated with LPS (red) or vehicle (blue). Asterisks indicate statistical differences between vehicle and LPS conditions. **(B)** Left: Absolute numbers of CD8^+^ T_CM_ cells expressing CD69 and CD38. Right: Absolute numbers of CD8^+^ T_EM/EFF_ cells expressing CD69 and CD38. LPS favors the increase of CD69^+^CD38^+^CD8^+^ T_EM/EFF_ cells in aged mice. Data is presented as mean ± S.D. Statistical significance was determined using an unpaired t-test with Welch’s correction. Significance levels are indicated as *p ≤ 0.05; **p ≤ 0.005. The data represent two different experiments (Veh, n = 4; LPS, n = 6, independent biological specimens).

Since CD38 is considered a marker of activated T cells (Motamedi et al., 2016), we are now investigating whether CD38 is co-expressed with CD69 in T cell subpopulations, which favors an activated phenotype in T cells from LPS-treated aged mice. Notably, there were greater proportions of CD69^+^ single-positive cells compared to CD38^+^ single-positive cells among the CD8^+^ T cells in unstimulated aged mice (Figure 4A). After LPS challenge, CD8^+^ T cells showed a significant increase in CD69^+^CD38^+^ double-positive activated phenotype in T_CM_ (*t* (5.356) = 6.074, *p* = 0.0014) and T_EFF/EM_ (*t* (7.938) = 2.979, *p* = 0.0178 cells compared to vehicle-treated mice (Figure 4B).

## 4 Discussion

CD38 plays a crucial role in promoting inflammation. Its expression and function increase with age, contributing to inflammaging and immunosenescence (Camacho-Pereira et al., 2016; Chini et al., 2018; Ghosh et al., 2023). Accordingly, our goal was to elucidate T cell bystander activation by the inflammatory challenge of LPS and to determine the necessity of CD38 for this inflammatory effect on memory T lymphocytes in an aged murine model. To achieve this, we focused on studying differentiation-defined subpopulations of T lymphocytes (naïve, central memory, and effector/effector memory cells) in response to acute LPS challenge.

The role of CD38 in inflammatory conditions varies depending on the model studied. Here, we report an increase in the inflammatory environment after LPS challenge in the presence of CD38. A previous report, using LPS administration as a sepsis model, demonstrated that CD38 promotes the expression of proinflammatory cytokines, including IL-1β, IL-6, and IL-12. That study also suggested that inhibiting CD38 could be a therapeutic strategy to delay the onset and progression of sepsis-induced acute kidney injury (Shu et al., 2018). However, others report that the presence of CD38 mediates the inhibition of NLRP3 inflammasome activation, thereby inhibiting IL-1β production and preventing endotoxemic lung injury (Farahany et al., 2022). In addition, in CD38 KO mice, there is increased TLR4 expression compared to WT mice, which favors the overexpression of IFN-γ and acute kidney injury (Li et al., 2019). These discrepancies may reflect differences in the cells and tissues analyzed, as well as the use of CD38 inhibition versus the KO mouse model. To evaluate the inflammatory effect of LPS, we measured the levels of IL-1β, IL-6, and IFN-γ. Since the data showed a higher inflammatory milieu in WT mice compared to CD38-deficient aged mice, even after the LPS challenge, we hypothesize that CD38 contributes to the elevation of serum cytokine levels following acute inflammation in aged mice and that CD38 deficiency protects aged mice from CD8^+^ T cell expansion after an acute proinflammatory challenge. However, the delta fold-change of IL-10 was comparable between WT and CD38-deficient aged mice, indicating that the regulatory IL-10 response is induced to a similar relative extent in both groups. In this context, the markedly higher delta induction of IL-1β, IL-6 and IFN-γ observed in aged WT mice is not counterbalanced by a proportionally greater increase in IL-10, potentially amplifying systemic inflammation. Although WT mice exhibit higher absolute IL-10 levels than CD38-deficient mice, the similar fold-change suggests that CD38 does not impair the capacity to induce IL-10 upon LPS exposure. Regulatory B and T cells are major producers of IL-10, and their suppressive function has been associated with CD38 expression (Domínguez-Pantoja et al., 2018; Pérez-Lara et al., 2021), raising the possibility that CD38 may influence the magnitude of regulatory responses. Altogether, these findings suggest that CD38 contributes to an unbalanced inflammatory milieu during aging by enhancing the proinflammatory response without a corresponding increase in the regulatory cytokine IL-10. Although previous reports support the production of these cytokines by T lymphocytes following LPS challenge (Piergallini et al., 2023), our experimental approach does not allow us to ascertain which cells produce these cytokines. Future experiments are needed to clarify the specific contribution of T cells to this non-specific proinflammatory phenotype and to assess the role of other immune cells.

Under basal conditions, our data indicate that CD38 promotes more differentiated cell states in aging, as evidenced by the higher numbers of CD44^hi^ cells in WT mice. Consistent with previous findings in aged mice, the proportion of naïve T cells is reduced, which is likely due to thymic involution and lifelong antigenic encounters, while the proportion of differentiated memory T cells is increased (Xia et al., 2022; Yang et al., 2021; Elyahu and Monsonego, 2021; Elyahu et al., 2019). Our observations suggest that CD38 may drive the transition from naïve to CD44-expressing cell phenotypes. Increasing evidence also suggests its potential role in inflammatory conditions and as a marker of T_RM_ cells (Evrard et al., 2023). Considering CD38 as a potential modulator of memory T cell differentiation during inflammation, it is essential to elucidate the possible mechanisms through which CD38 influences age-associated inflammation T cell fitness.

Reports have shown that the presence of cytokines, such as IL-2, is necessary for the activation and differentiation of naïve T cells into the memory phenotype. Altered IL-2 production has been reported in aged mice (Adolfsson et al., 2001; Raynor et al., 2013). Additionally, a study conducted by our group indicates a positive correlation between CD38 and the expression of CD25 (IL-2Rα) in regulatory T lymphocytes (Pérez-Lara et al., 2021). According to the results described here, the absence of CD38 might compromise the production of IL-2 or the expression of its receptors on T cells. These results may explain why aged KO mice retain a CD44^lo^ T cell phenotype in contrast to WT mice. The increased T_EFF/EM_ phenotype found in WT aged mice raises the question of whether CD38 is actively modulating T cell differentiation or whether its effect on inflammatory response affects T cell homeostasis throughout the lifespan.

Our findings also highlight the role of CD38 in the response to LPS-induced CD8^+^ T cell responses. Previous work has shown that LPS can promote T cell antigen-independent proliferation of CD8^+^CD44^hi^ T cells (Tough et al., 1997). Due to the inflammatory milieu present in aging, our results suggest that CD38 regulation may abrogate the LPS-induced changes observed in CD8^+^ T cell subpopulations in aging. These findings point to a more nuanced role for CD38 in non-specific T cell activation during age-related inflammatory diseases, where CD8^+^ T cell subsets regulate immune responses.

Antigen-independent memory T cells (AIMT) or virtual memory T cells are cells that exhibit a T_CM_-like phenotype. Our results demonstrate an overrepresentation of CD8^+^ CD44^hi^ cells in CD38-bearing mice. The selective increase of CD69^+^ and Ki67^+^ cells within the CD8^+^ T_CM_ compartment likely reflects the expansion of AIMT cells. These cells are present in mice that have not been exposed to antigens. They accumulate during aging and are susceptible to bystander activation, mainly mediated by the presence of IL-15 and IL-4, rather than antigen-specific TCR stimulation (Renkema et al., 2014; White et al., 2016). This could explain why not only activation but also proliferation markers are preferentially observed in T_CM_-like populations rather than in the T_EFF/EM_ subset. However, it is necessary to evaluate alterations in the production of IL-4, IL-15, and CD122 expression, and to include markers that allow discrimination between AIMT and accurate memory phenotypes, such as CD49d. Overall, our data support a CD38-dependent T cell phenotype in the aging process.

CD38 is a marker of activation in aging and inflammation in adipose tissue, liver, and innate immune cells such as macrophages (Covarrubias et al., 2020; Chini et al., 2020). Here, we observed increased CD38 expression on T_EFF/EM_ lymphocytes following LPS-induced inflammation. Among Toll-like receptor agonists, LPS is one of the most potent inducers of CD38, particularly in macrophages and other innate immune cells. Additionally, in aging, persistent exposure to microbial products and cytokines such as IL-6, IL-10, and TNF-α—components of the senescence-associated secretory phenotype (SASP)—further enhances CD38 expression. This highlights the interplay between CD38 and the inflammatory milieu, with the increase of proinflammatory cytokines occurring in a CD38-dependent manner, and the induction of CD38 by this inflammatory environment. In addition, to evaluate the activation state of aged KO mice, we measured CD69, a marker of T lymphocyte activation. LPS challenge increased the number of T_CM_ CD69^+^CD8^+^ T lymphocytes in a CD38-dependent manner. These findings align with previous reports, showing a positive correlation between CD38 and CD69, among other regulatory markers on regulatory T cells (Pérez-Lara et al., 2021). Prior work described CD69^+^ CD8^+^ T cell dysregulated activation after the combination of *in vitro* TCR activation and LPS, highlighting the potential contribution to organ dysfunction (Piergallini et al., 2023). Although we did not directly assess CD38 expression following LPS stimulation, previous work by Tincati et al. (2012) demonstrated that stimulation of PBMCs with LPS increased CD38 expression on CD8^+^ T cells in both healthy individuals and HIV-infected patients receiving antiretroviral therapy. This finding suggests that CD38 upregulation in response to inflammatory stimuli also occurs in human CD8^+^ T cells, consistent with our observations in mice (Tincati et al., 2012). Interestingly, in HIV-positive individuals, a phenomenon resembling inflammaging has been described, highlighting striking parallels between chronic viral infection and age-associated immune activation (Rod es et al., 2022).

Moreover, recent studies have demonstrated that CD38 expression can contribute to T cell exhaustion and diminished cytotoxicity in various pathological contexts. For instance, CD38 has been identified as a co-exhaustion marker in CD8^+^ tissue-resident memory T cells in hepatocellular carcinoma (HCC), correlating with increased PD-1 expression and higher histopathological grades (Reolo et al., 2023). Additionally, CD38 inhibition has been shown to enhance the anti-tumor function of chimeric antigen receptor (CAR) T cells, suggesting its role in T cell dysfunction. These findings underscore the need to investigate the role of CD38 in regulating T cell function in the elderly (Veliz et al., 2024).

Our data support the idea that CD38 may promote the increase of bystander-activated T cells through the proinflammatory milieu induced by LPS challenge. Given that CD38 is overexpressed during aging in both mice and humans (Camacho-Pereira et al., 2016), it would be essential to define its role in non-specific T cell activation in inflammation-related diseases of the elderly.

NAD^+^ metabolism plays a central role during T cell activation, proliferation, and effector function. Through immune responses, activated T cells rely on adequate NAD^+^ availability to sustain the high metabolic demands required for cytokine production, mitochondrial activity, and biosynthetic processes (Camacho-Pereira et al., 2016). However, in environments where NAD^+^ becomes a limiting resource, such as in the tumor microenvironment or during chronic inflammation, it affects T cell activity. Studies have shown that low intracellular NAD^+^ levels suppress T cell proliferation and cytotoxic function (Sh et al., 2025). At the same time, exogenous NAD^+^ precursors, such as nicotinamide (NAM), can restore their effector capacity through pathways that include STING axis activation and improved mitochondrial metabolism (Sh et al., 2025).

As a major NADase, CD38 overexpression in T cells may exacerbate NAD^+^ depletion, leading to metabolic exhaustion and impaired immune function. Elevated CD38 activity reduces intracellular NAD^+^ pools, limiting the availability of this essential cofactor for energy metabolism and signaling pathways necessary for T cell activation. Consequently, persistent CD38 upregulation could lead to T cell dysfunction by promoting a state of metabolic insufficiency and reduced anti-tumor or antiviral capacity (Camacho-Pereira et al., 2016).

The balance between CD38 expression and NAD^+^ availability represents a potential therapeutic target to restore immune competence in aged or chronically inflamed environments. Inhibiting CD38 could help preserve intracellular NAD^+^ pools, thereby improving mitochondrial metabolism, energy production, and T cell functionality during aging. Moreover, combining CD38 blockade with NAD^+^ precursor supplementation may offer a synergistic therapeutic approach to restore metabolic homeostasis, mitigate immunosenescence, and counteract chronic inflammation associated with age-related diseases (Camacho-Pereira et al., 2016; Yu et al., 2025).

Our results indicate that CD38 promotes bystander activation of memory T cells in the context of inflammation in aged mice. These results are supported by our analysis of proliferating T lymphocyte subpopulations following LPS administration, which revealed a reduction in Ki67^+^CD8^+^ T_CM_ lymphocytes in CD38-deficient mice.

We also report here that LPS promotes the simultaneous expression of CD38 and CD69 in T_EFF/EM_ CD8^+^ cells from aged LPS-challenged WT mice, which likely represents a distinct activation state from T_CM_ CD69 single-positive cells. This result suggests that CD38 drives a highly activated effector phenotype in response to inflammation. However, the function of CD38^+^CD69^+^ T_EFF/EM_ cells in this context remains unclear, and their strong activation may be detrimental, contributing to organ damage in uncontrolled inflammation (59).

## 5 Conclusion

Here, we describe the protein CD38 as a driver of bystander activation of memory T cells, promoting the proliferation of T_CM_ and highly activated T_EFF/EM_ cells in aged mice. Our findings demonstrate that the effects of CD38 on LPS-induced inflammation extend beyond innate immune cells, also influencing memory T cell responses. We also highlight the CD38^−^CD69 axis and the potential contribution to both activation and homeostasis of CD8^+^ T cells during aging. Together, these results position CD38 as a key regulator of adaptive immunity in age-related inflammatory diseases, such as autoimmunity, cancer, and inflammation-driven immune dysfunction.

## 6 Limitations of the study

However, several limitations should be considered. First, we did not directly validate our findings in human cells, which would strengthen the translational relevance of our results. Additionally, although we measured systemic cytokine levels, the cellular sources and precise contributions of specific T cell subsets remain unclear. Further mechanistic studies, including intracellular cytokine profiling, proteomic and metabolomic analyses, and transcriptomic approaches such as RNA sequencing, would provide a more comprehensive understanding of how CD38 regulates T cell metabolism, activation, and effector function during aging, as well as the potential role of sex bias. Incorporating these approaches in future work could elucidate the pathways and molecular networks influenced by CD38, thereby clarifying its role in immunosenescence and age-associated inflammatory diseases. Finally, although the experimental groups were small (n = 4–6), the results showed consistent trends across independent experiments and reached statistical significance for key outcomes. Future studies with larger cohorts are warranted to confirm and extend these findings.

Together, despite these limitations, our results position CD38 as a key regulator of adaptive immunity in age-related inflammatory diseases, such as autoimmunity, cancer, and inflammation-driven immune dysfunction.

## Data Availability

The raw data supporting the conclusions of this article will be made available by the authors, without undue reservation.

## Supplementary material

SUPPLEMENTARY FIGURE 1 Serum cytokines in response to acute LPS challenge in aged mice. A) Bars represent the mean change (Δ) in serum cytokine concentration calculated as *mean (LPS) – mean (Vehicle)* for each group. Error bars denote the standard error of the mean (SEM). Data were analyzed using a one-way ANOVA followed by Tukey’s test. (Veh, n = 4; LPS, n = 6, independent biological specimens). **p ≤ 0.005; ***p ≤ 0.001; ****p ≤ 0.0001.

SUPPLEMENTARY FIGURE 2 CD8^+^ T cell subpopulations flow gating strategy and TLR4 expression in CD4^+^ and CD8^+^ T lymphocytes. A) 2 × 10^6^ total splenocytes were stained, FSC-A versus FSC-H discriminated against doublets (debris and dead cells) from singlets. We then evaluated size versus granularity (FSC-A versus SSC-A) and selected small, non-granular splenocytes that matched the description of lymphocytes. We then assessed the expression of CD19 protein, a marker for B lymphocytes, and selected the CD19-negative cells, which were found to be predominantly T-lymphocytes, accounting for 95% of the cells. From the CD19-negative region, we selected CD8^+^ cells and assessed the expression of CD44 and CD62L proteins, which allowed us to identify Naïve (CD44^neg^CD62L^hi^), T_CM_ (CD44^hi^CD62L^hi^), T_EFF/EM_ (CD44^hi^CD62L^low^) cells. B) percentage (left) and absolute numbers (right) of T_CM_ and T_EFF/EM_ from KO LPS challenged mice. No statistical differences were found. C) Total TLR4^+^ CD4^+^ and D) CD8^+^ T cells were evaluated in WT and KO aged mice. Percentages of T cell subpopulations were analyzed using a two-way ANOVA followed by Tukey’s test. Data are presented as mean ± S.D. *p ≤ 0.05; ****p ≤ 0.0001.The data represent two different experiments (Veh, n = 4; LPS, n = 6, independent biological specimens). TLR4 data were analyzed using a one-way ANOVA followed by Tukey’s test. Data represented as mean ± S.D. **p ≤ 0.005; ***p ≤ 0.001. Flow cytometry panel B was used to determine CD4^+^TLR4^+^ and CD8^+^TLR4^+^ T cells (veh, n = 4, LPS, n = 8, independent biological specimens).

SUPPLEMENTARY FIGURE 3 CD69^+^ and Ki67^+^ T_CM_ CD8^+^ T cells are increased in LPS-challenged aged mice. A) Fold delta change of CD69^+^ T_CM_ CD8^+^ T cells in LPS-challenged mice according to baseline conditions (left). Fold delta change of CD69 MFI gated on T_CM_ CD8^+^ T cells in LPS-challenged mice according to baseline conditions (right). B) Fold delta change of CD69^+^ T_EFF/EM_ CD8^+^ T cells in LPS-challenged mice according to baseline conditions (left). Fold delta change of CD69 MFI gated on T_EFF/EM_ CD8^+^ T cells in LPS-challenged mice according to baseline conditions (right). C) Fold delta change of Ki67^+^ T_CM_ CD8^+^ T cells in LPS-challenged mice according to baseline conditions (left). Fold delta change of Ki67^+^ T_EFF/EM_ CD8^+^ T cells in LPS-challenged mice according to baseline conditions (right). Statistical significance was determined using an unpaired two-tailed Student’s t-test. Data are presented as mean ± S.D. Significance levels are indicated as *p ≤ 0.05 or the exact p value.

SUPPLEMENTARY FIGURE 4 Fluorescence Minus One (FMO) control. FMO controls were performed using cells from two WT LPS samples randomly chosen and stained with all antibodies included in our panel, except for either CD69 or CD38. CD69 FMO was stained with the entire panel without the CD69-PE antibody. For the CD38 FMO control, we stained the cells with the entire panel without the CD38-FITC antibody.
