# Supplementary figures and images for: CD38 promotes LPS-induced innate-like activation and proliferation of CD8+ T lymphocytes in aged mice
Source: Front Aging. 2025 Dec 19;6:1701685. doi: 10.3389/fragi.2025.1701685 (PMC12757697; doi:10.3389/fragi.2025.1701685)

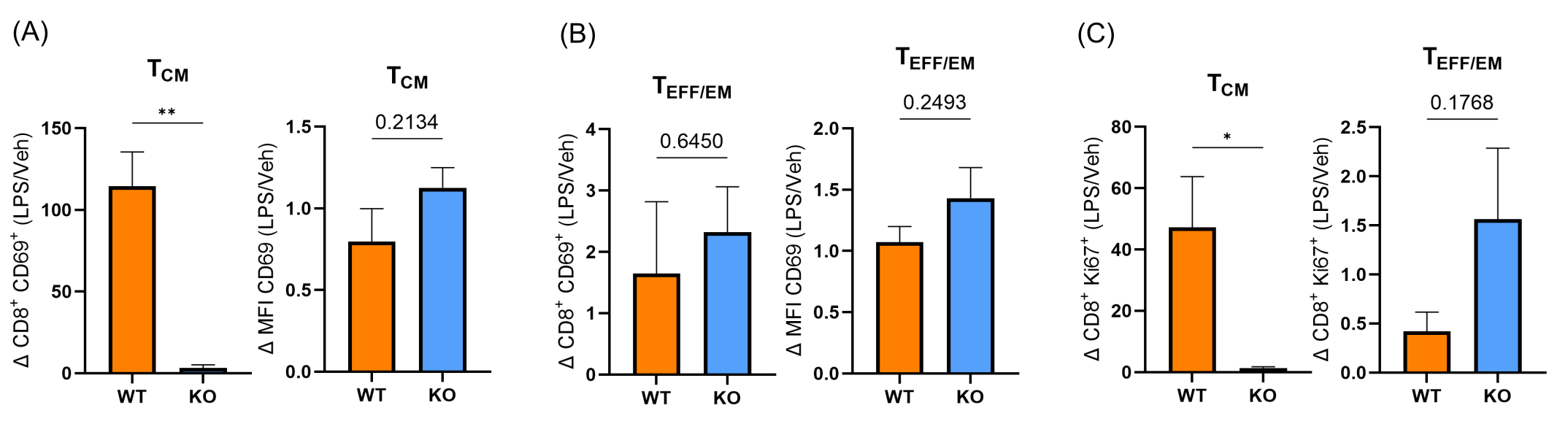

Supplement: Supplementary file 1 [file Image3.tiff]

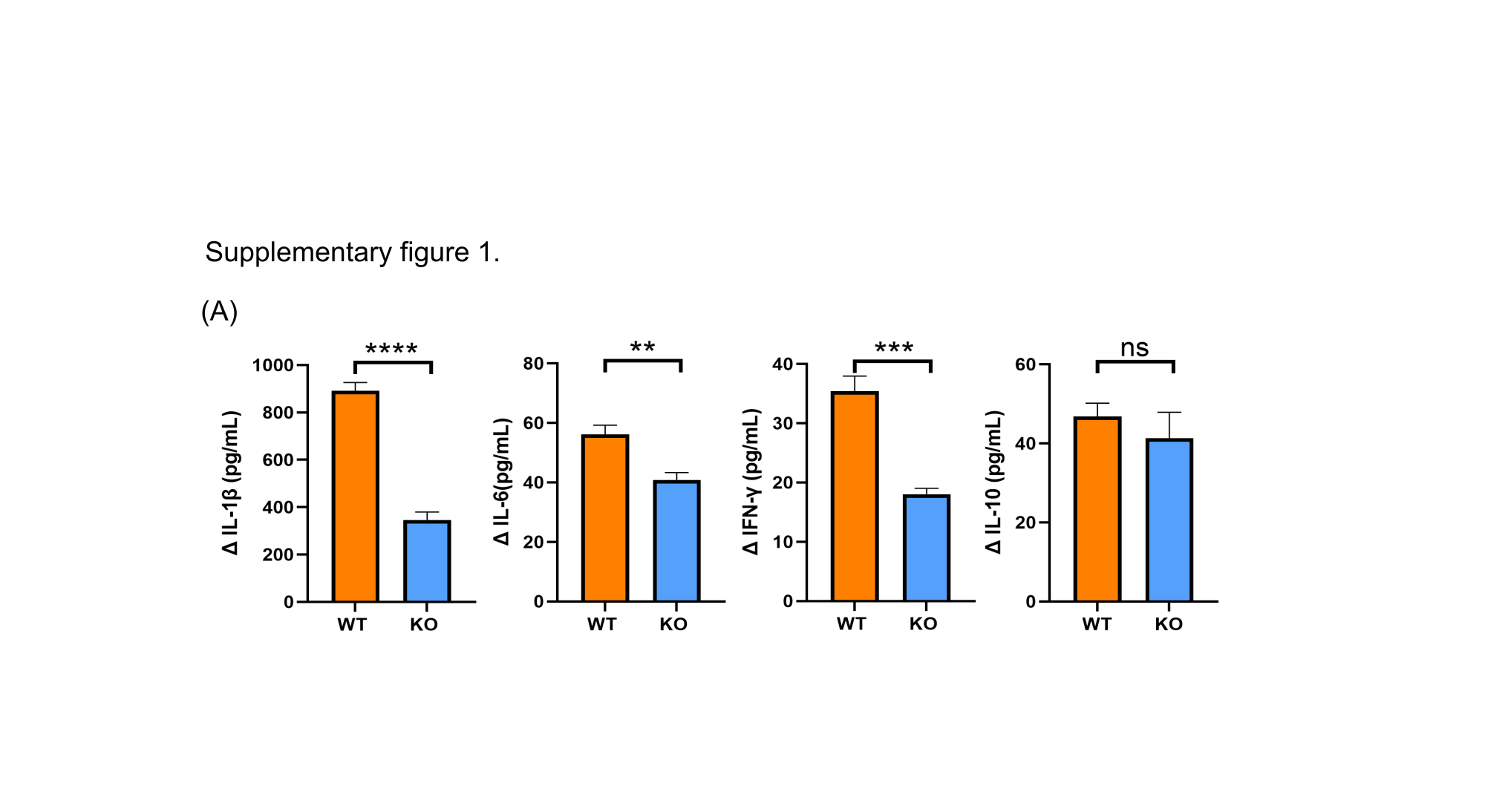

Supplement: Supplementary file 2 [file Image1.tiff]

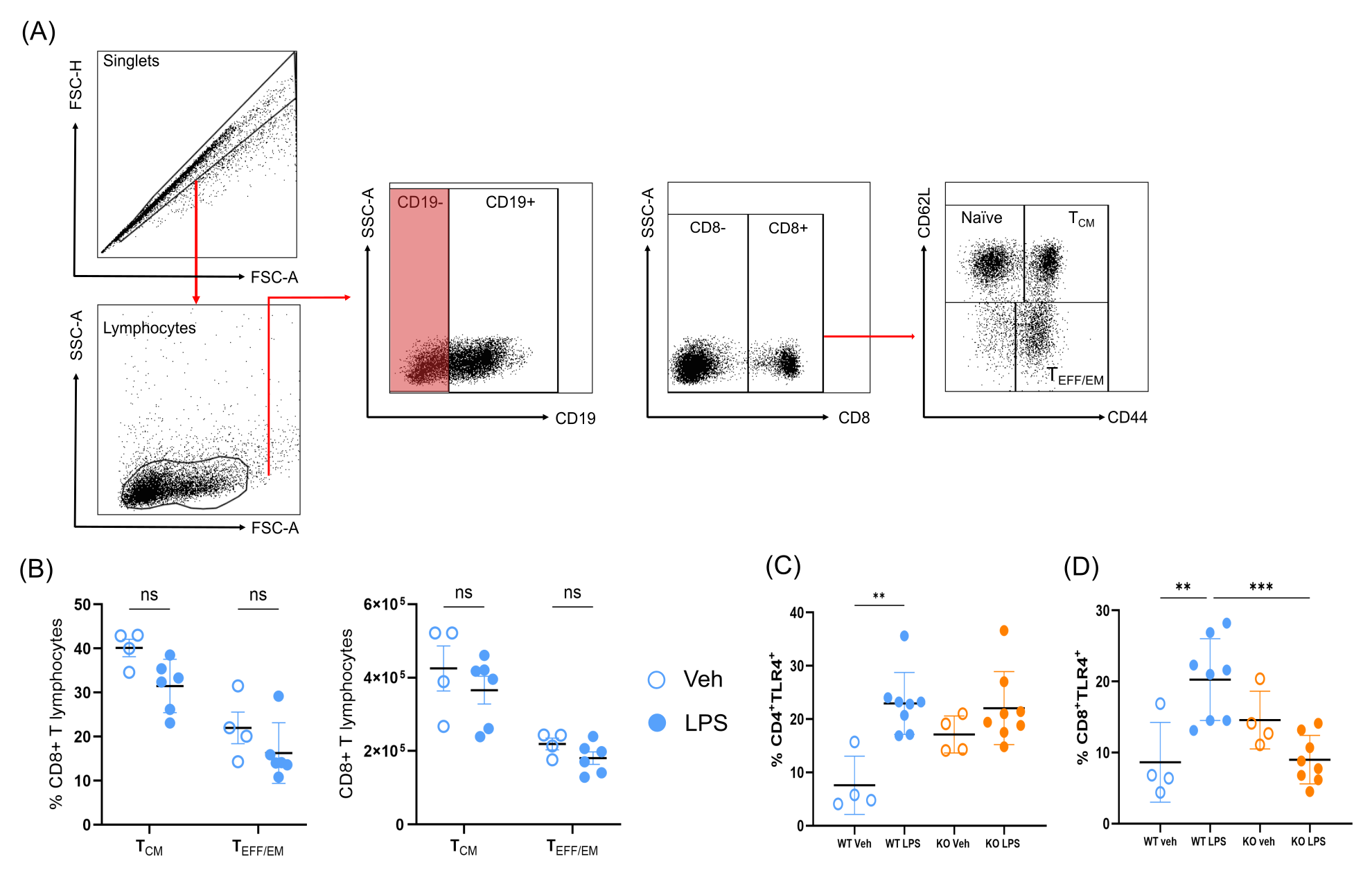

Supplement: Supplementary file 7 [file Image2.tiff]

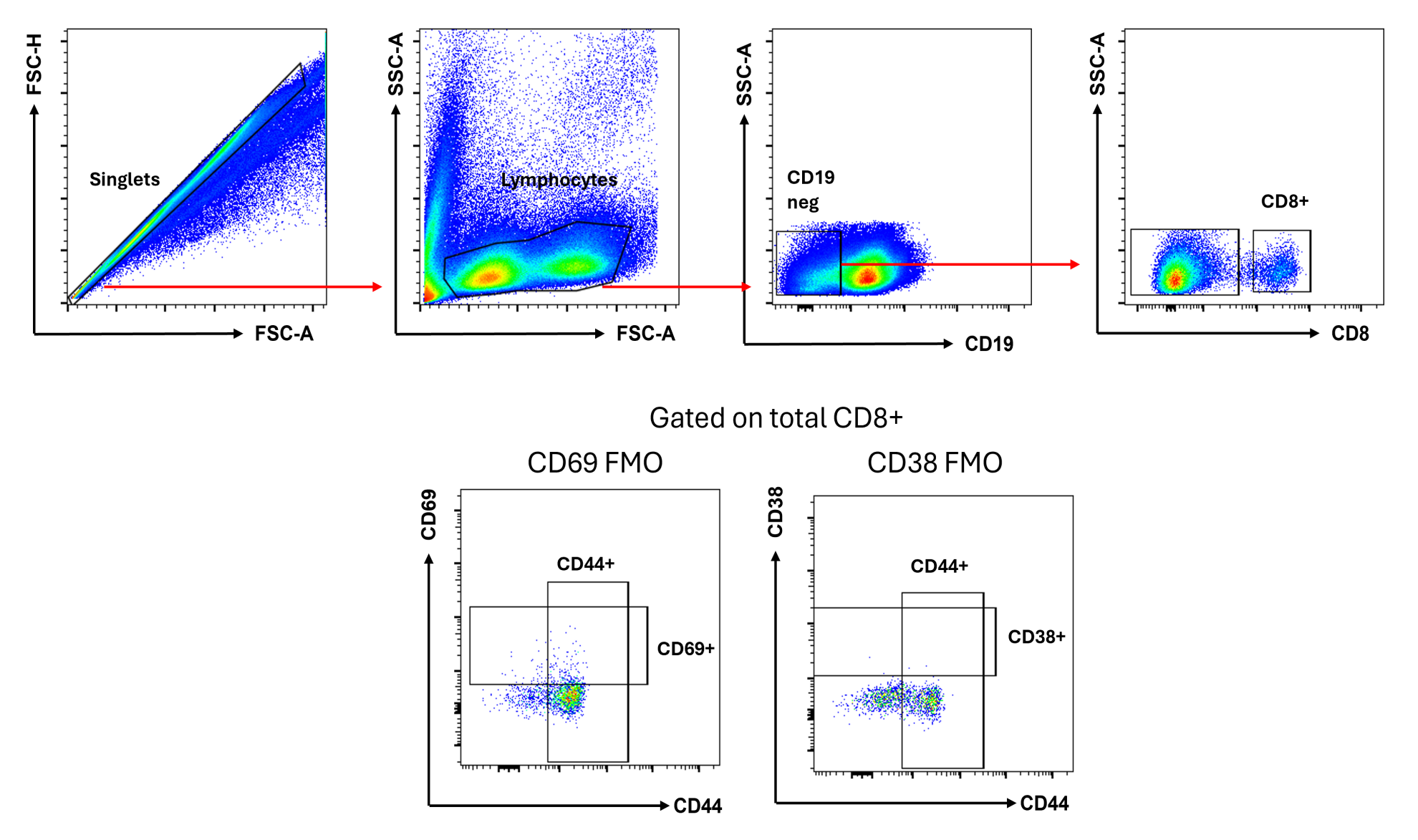

Supplement: Supplementary file 8 [file Image4.tiff]
