# Supplementary material for: CD38 promotes LPS-induced innate-like activation and proliferation of CD8+ T lymphocytes in aged mice
Source: Front Aging. 2025 Dec 19;6:1701685. doi: 10.3389/fragi.2025.1701685 (PMC12757697; doi:10.3389/fragi.2025.1701685)
Supplement: Supplementary file 3 [file Table1.docx]

## **Supplementary Table 1.** Cytokine levels and spleen parameters after LPS stimulation in wild-type and CD38 knockout mice.

| Outcome | Subset | Model | Groups / Descriptives (mean ± SEM, n) | Statistic | p (adjusted) | Effect size [95% CI] | Assumptions |
| --- | --- | --- | --- | --- | --- | --- | --- |
| IL-1β | Total serum levels | Two-way ANOVA (Genotype × LPS) | WT veh= 461.75 ± 20.27 (4); WT LPS = 1353.83 ± 27.622 (6); KO veh = 98.5 ± 6.03 (4); KO LPS = 444.5 ± 32.86 (6) | F (1, 16) = 94.38 | Veh:KO vs. Veh:WT p= <0.0001; LPS:WT vs. Veh:WT p= <0.0001; LPS:KO vs. Veh:WT p= 0.9717; LPS:WT vs. Veh:KO p= <0.0001; LPS:KO vs. Veh:KO p= <0.0001; LPS:KO vs. LPS:WT p= <0.0001 | 426.9 to 665.2 | Shapiro p = 0.1451 |
| IL-6 | Total serum levels | Two-way ANOVA (Genotype × LPS) | WT veh= 45.75 ±2.21 (4); WT LPS = 101.83 ±2.22 (6); KO veh = 17.5 ± 0.64 (4); KO LPS = 58.33 ± 2.36 (6) | F (1, 16) = 11.75 | Veh:KO vs. Veh:WT p= <0.0001; LPS:WT vs. Veh:WT p= <0.0001; LPS:KO vs. Veh:WT p= 0.0051; LPS:WT vs. Veh:KO p= <0.0001; LPS:KO vs. Veh:KO p= <0.0001; LPS:KO vs. LPS:WT p= <0.0001 | 5.820 to 24.68 | Shapiro p = 0.9708 |
| IFN-γ | Total serum levels | Two-way ANOVA (Genotype × LPS) | WT veh= 59.75 ± 1.43 (4); WT LPS = 95.16 ±2.08 (6); KO veh = 17.5 ± 0.64 (4); KO LPS = 35.5 ±0.80 (6) | F (1, 16) = 32.34 | Veh:KO vs. Veh:WT p= <0.0001; LPS:WT vs. Veh:WT p= <0.0001; LPS:KO vs. Veh:WT p= 0.0001; LPS:WT vs. Veh:KO p= <0.0001; LPS:KO vs. Veh:KO p= <0.0001; LPS:KO vs. LPS:WT p= <0.0001 | 10.92 to 23.91 | Shapiro p = 0.9520 |
| IL-10 | Total serum levels | Two-way ANOVA (Genotype × LPS) | WT veh= 42.25 ± 1.31 (4); WT LPS = 87.5 ± 1.17 (6); KO veh = 15.5 ± 1.44 (4); KO LPS = 55.5 ± 2.17 (6) | F (1, 16) = 2.313 | Veh:KO vs. Veh:WT p= 0.6450; LPS:WT vs. Veh:WT p= 0.0014; LPS:KO vs. Veh:WT p= 0.0214; LPS:WT vs. Veh:KO p= <0.0001; LPS:KO vs. Veh:KO p= 0.0010; LPS:KO vs. LPS:WT p= 0.4684 | -2.067 to 12.57 | Shapiro p = 0.3275 |
| Spleen weight | Spleen weight (grams) | Two-way ANOVA (Genotype × LPS) | WT veh= 0.095 ± 0.19 (4); WT LPS = 0.2 ± 0.17 (8); KO veh = 0.063 ± 0.005 (4); KO LPS = 0.171 ± 0.012 (8) | F (1, 20) = 0.01239 | Veh:KO vs. Veh:WT p= <0.0001; LPS:WT vs. Veh:WT p= <0.0001; LPS:KO vs. Veh:WT p= 0.0003; LPS:WT vs. Veh:KO p= <0.0001; LPS:KO vs. Veh:KO p= <0.0001; LPS:KO vs. LPS:WT p= <0.0001 | -0.07402 to 0.06652 | Shapiro p = 0.5430 |
| Cell count | Total splenocytes | Two-way ANOVA (Genotype × LPS) | WT veh= 4.57e+007 ± 6249716.6 (4); WT LPS = 9.49e+007 ± 1.49e+007 (8); KO veh = 2.9375e+007 ± 1360376.78 (4); KO LPS = 1.07e+008 ± 2.083e+007 (8) | F (1, 20) = 0.6101 | Veh:KO vs. Veh:WT p= 0.9491; LPS:WT vs. Veh:WT p= 0.2782; LPS:KO vs. Veh:WT p= 0.1222; LPS:WT vs. Veh:KO p= 0.0950; LPS:KO vs. Veh:KO p= 0.0358; LPS:KO vs. LPS:WT p= 0.9326 | -107362426 to 48862426 | Kolmogorov-Smirnov (distance) p = 0.0659 |
| Cell count | Total CD3+ T cells | Two-way ANOVA (Genotype × LPS) | WT veh= 7334040.01 ± 1024775.78 (4); WT LPS = 1.33e+007± 1721781.81 (8); KO veh = 5535036 ± 852021.40 (4); KO LPS = 1.34e+007 ±2967966.77 (8) | F (1, 20) = 0.1379 | Veh:KO vs. Veh:WT p= 0.9715; LPS:WT vs. Veh:WT p= 0.3510; LPS:KO vs. Veh:WT p= 0.3404; LPS:WT vs. Veh:KO p= 0.1572; LPS:KO vs. Veh:KO p= 0.1512; LPS:KO vs. LPS:WT p= >0.9999 | -12414826 to 8661996 | Kolmogorov-Smirnov (distance) p = 0.1000 |
| Cell count | Total CD4+ T cells | Two-way ANOVA (Genotype × LPS) | WT veh= 2858670.25 ± 539376.80 (4); WT LPS = 3800102.25± 581573.817 (8); KO veh = 1682103 ± 350477.52(4); KO LPS = 3491837.75 ±885772.91 (8). | F (1, 20) = 0.7459 | Veh:KO vs. Veh:WT p= 0.4093; LPS:WT vs. Veh:WT p= 0.8034; LPS:KO vs. Veh:WT p= 0.9908; LPS:WT vs. Veh:KO p= 0.0572; LPS:KO vs. Veh:KO p= 0.1795; LPS:KO vs. LPS:WT p= 0.8777 | -0.03268 to 0.3438 | Shapiro p =0.2006 |
| Cell count | Total CD8+ T cells | Two-way ANOVA (Genotype × LPS) | WT veh= 2598110 ± 333752.91 (4); WT LPS = 5583043.5± 731715.29(8); KO veh = 2767713.5 ± 627869.53(4); KO LPS = 5899580.875 ±1041561.24 (8). | F (1, 20) = 0.005947 | Veh:KO vs. Veh:WT p= 0.9995; LPS:WT vs. Veh:WT p= 0.1531; LPS:KO vs. Veh:WT p= 0.0995; LPS:WT vs. Veh:KO p= 0.1905; LPS:KO vs. Veh:KO p= 0.1258; LPS:KO vs. LPS:WT p= 0.9914. | -4121264 to 3827396 | Shapiro p = 0.2925 |

A Two-way ANOVA test was applied to evaluate serum cytokine levels (IL-1β, IL-6, IFN-γ, IL-10), spleen weight, and splenocyte/T cell counts between genotypes (WT, CD38-KO) and treatments (vehicle, LPS). CD4+ T total cell counts were analyzed on log-^10^-transformed data to meet the normality assumptions. Data are expressed as mean ± SEM. Normality was assessed using the Shapiro–Wilk or Kolmogorov–Smirnov tests.
