# Supplementary material for: CD38 promotes LPS-induced innate-like activation and proliferation of CD8+ T lymphocytes in aged mice
Source: Front Aging. 2025 Dec 19;6:1701685. doi: 10.3389/fragi.2025.1701685 (PMC12757697; doi:10.3389/fragi.2025.1701685)
Supplement: Supplementary file 4 [file Table2.docx]

**Supplementary Table 2.** Proportion and counts of CD8⁺ T cell subsets following LPS exposure in wild-type and CD38 knockout mice.

| Outcome | Subset | Model | Groups / Descriptives (mean ± SEM, n) | Statistic | p (adjusted) | Effect size [95% CI] | Assumptions |
| --- | --- | --- | --- | --- | --- | --- | --- |
| % CD8+ T cells | T_CM__T_EFF/EM_ | Two-way ANOVA (Genotype × LPS) | CD44lo: WT veh = 33 ± 8.57 (4); WT LPS = 42.68 ± 3.459(6); KO veh = 38 ± 1.87(4); KO LPS = 52.5 ± 4.27(6). CD44hi: WT veh = 66.25 ± 8.57 (4); WT LPS = 52.05 ± 5.28 (6); KO veh = 62 ± 1.87(4); KO LPS = 47.66 ± 4.43 (6). | F (3, 32) = 5.241 | CD44lo: WT veh vs. CD44hi: WT veh p = 0.0071. | - 22.96 to -7.929 | D'Agostino-Pearson omnibus (K2) = 0.6517 |
| CD8+ T cell numbers | T_CM__T_EFF/EM_ | Two-way ANOVA (Genotype × LPS) | CD44lo: WT veh = 847929.75 ± 191336.43 (4); WT LPS = 926660.66 ± 154956.04 (6); KO veh = 402888.5 ± 57533.72 (4); KO LPS = 646541.83 ± 112633.10 (6). CD44hi: WT veh = 2145255.25 ± 835292.88 (4); WT LPS = 1160479.83 ± 181688.14 (6); KO veh = 644355.25 ± 55548.44 (4); KO LPS = 546685 ± 44240.31 (6). | F (3, 32) = 1.792 | WT veh CD44lo vs. CD44hi p = 0.0147; CD44hi WT veh vs. KO veh p = 0.0145; CD44hi WT LPS vs. KO LPS p = 0.0365 | - 0.2824 to -0.03301 | “Non-normal distribution Log-10 -transformed data analysis applied" |

Statistical comparison of CD44lo (TCM) and CD44hi (TEFF/EM) CD8⁺ T cell subsets between genotypes and treatments. Data analyzed by Two-way ANOVA presented as mean ± SEM. The D’Agostino–Pearson and Shapiro–Wilk tests were used to assess normality.
