# Supplementary material for: CD38 promotes LPS-induced innate-like activation and proliferation of CD8+ T lymphocytes in aged mice
Source: Front Aging. 2025 Dec 19;6:1701685. doi: 10.3389/fragi.2025.1701685 (PMC12757697; doi:10.3389/fragi.2025.1701685)
Supplement: Supplementary file 5 [file Table3.docx]

**Supplementary Table 3.** Activation and proliferation markers in CD8⁺ T cell subsets after LPS stimulation in wild-type and CD38 knockout mice.

| Outcome | Subset | Model | Groups / Descriptives (mean ± SEM) | Statistic | p (adjusted) | Effect size [95% CI] | Assumptions |
| --- | --- | --- | --- | --- | --- | --- | --- |
| Cell count | T_CM_ CD8^+^CD38^+^ | Unpaired t-test with Welch's correction | WT veh = 1851 ± 955.8; WT LPS = 63562 ± 10022 | t = 6.130, df = 5.091 | WT LPS vs WT veh p = 0.0015 | 35969 to 87454 | Shapiro-Wilk (W) p = 0.3103 |
| Cell count | T_EFF/EM_ CD8^+^CD38+ | Unpaired t-test with Welch's correction | WT veh = 68342 ± 16813; WT LPS = 150940 ± 54166 | t = 1.456, df = 5.918 | WT LPS vs WT veh p = 0.1962 | - 56645 to 221842 | Unpaired t-test with Welch's correction |
| CD38 MFI | T_CM_ CD8^+^ | Unpaired t-test with Welch's correction | WT veh = 118.8 ± 3.326; WT LPS = 117.0 ± 1.713 | t = 0.4678, df = 4.608 | WT LPS vs WT veh p = 0.3451 | - 11.62 to 8.119 | Shapiro-Wilk (W) p = 0.7817 |
| CD38 MFI | T_EFF/EM_ CD8^+^ | Unpaired t-test with Welch's correction | WT veh = 129.8 ± 9.827; WT LPS = 170.8 ± 12.18 | t = 2.625, df = 7.988 | WT LPS vs WT veh p = 0.5233 | 4.980 to 77.19 | Shapiro-Wilk (W) p = 0.3103 |
| CD69+ | T_CM_ CD8^+^ | Welch's ANOVA test (Genotype × LPS) | WT veh = 2505 ± 1126 (4); WT LPS = 201300 ± 41290 (6); KO veh = 52613 ± 21439 (4); KO LPS = 96291 ± 14464 (6) | W = 19.57 (3.000, 6.860) p = 0.0010 | WT veh vs. WT LPS p = 0.0223; WT veh vs. KO veh p = 0.3313; WT veh vs. KO LPS p = 0.0062; WT LPS vs. KO veh p = 0.0733; WT LPS vs. KO LPS p = 0.2261; KO veh vs. KO LPS p = 0.5093. | 0.837 | Shapiro-Wilk (W) p = 0.1121 |
| CD69+ | T_EFF/EM_ CD8^+^ | Welch's ANOVA test (Genotype × LPS) | WT veh = 274769 ± 103920 (4); WT LPS = 183295 ± 58593 (6); KO veh = 34267 ± 7535 (4); KO LPS = 35640 ± 7451 (6) | W = 5.558 (3.000, 7.515) p = 0.0257 | WT veh vs. WT LPS p = 0.9550; WT veh vs. KO veh p = 0.3382; WT veh vs. KO LPS p = 0.4373; WT LP vs. KO veh p = 0.2157; WT LPS vs. KO LPS p = 0.3974; KO veh vs. KO LPS p = 0.0763. | 0.543 | “Non-normal distribution" |
| CD69 MFI | T_CM_ CD8^+^ | Welch's ANOVA test (Genotype × LPS) | WT veh = 217.3 ± 27.29 (4); WT LPS = 165.5 ± 16.88 (6); KO veh = 147.8 ± 12.50 (4); KO LPS = 174.8 ± 13.87 (6) | W = 1.733 (3.000, 8.027) p = 0.2371 | WT veh vs. WT LPS p = 0.5583; WT veh vs. KO veh p = 0.2975; WT veh vs. KO LPS p = 0.6806; WT LPS vs. KO veh p = 0.9393; WT LPS vs. KO LPS p = 0.9980; KO veh vs. KO LPS p = 0.6351. | 0.155 | Shapiro-Wilk (W) p = 0.1155 |
| CD69 MFI | T_EFF/EM_ CD8^+^ | Welch's ANOVA test (Genotype × LPS) | WT veh = 183.3± 25.16 (4); WT LPS = 193.5 ± 9.922 (6); KO veh = 159.3 ± 11.26 (4); KO LPS = 215.5 ± 18.30 (6) | W = 2.478 (3.000, 7.799) p = 0.1374 | WT veh vs. WT LPS p = 0.9984; WT veh vs. KO veh p = 0.9215; WT veh vs. KO LPS p = 0.8627; WT LPS vs. KO veh p = 0.2454; WT LPS vs. KO LPS p = 0.8569; KO veh vs. KO LPS p = 0.1457. | 0.273 | Shapiro-Wilk (W) p = 0.1689 |
| Cell count | T_CM_ Ki67^+^ | Welch's ANOVA test (Genotype × LPS) | WT veh = 1378 ± 1110 (4); WT LPS = 15006 ± 2093 (6); KO veh = 5414 ± 575.8 (4); KO LPS = 6682 ± 1133 (6) | W = 10.19 (3.000, 8.329) p = 0.0037 | WT veh vs. WT LPS p = 0.0037; WT veh vs. KO veh p = 0.1012; WT veh vs. KO LPS p = 0.0515; WT LPS vs. KO veh p = 0.0219; WT LPS vs. KO LPS p = 0.0415; KO veh vs. KO LPS p = 0.8817. | 0.691 | Shapiro-Wilk (W) p = 0.2979 |
| Cell count | T_EFF/EM_ Ki67^+^ | Welch's ANOVA test (Genotype × LPS) | WT veh = 107007 ± 65344 (4); WT LPS = 22561 ± 3547 (6); KO veh = 13771 ± 4247 (4); KO LPS = 12018 ± 2284 (6) | W = 2.330 (3.000, 7.028) p = 0.1605 | WT veh vs. WT LPS p = 0.7364; WT veh vs. KO veh p = 0.6728; WT veh vs. KO LPS p = 0.6591; WT LPS vs. KO veh p = 0.5573; WT LPS vs. KO LPS p = 0.1622; KO veh vs. KO LPS p = 0.9989. | 0.266 | “Non-normal distribution” |

Statistical analysis of CD38⁺, CD69⁺, and Ki67⁺ expression in T_CM_ and _TEFF/EM_ CD8⁺ T cells under vehicle or LPS treatment. Welch’s ANOVA and Unpaired t-test with Welch correction were applied according to data distribution. Values represent mean ± SEM.
