# Supplementary material for: CD38 promotes LPS-induced innate-like activation and proliferation of CD8+ T lymphocytes in aged mice
Source: Front Aging. 2025 Dec 19;6:1701685. doi: 10.3389/fragi.2025.1701685 (PMC12757697; doi:10.3389/fragi.2025.1701685)
Supplement: Supplementary file 6 [file Table4.docx]

## **Supplementary Table 4.** Statistical analysis of CD38⁺CD69⁺ T cell subsets after LPS exposure in wild-type mice.

| Outcome | Subset | Model | Groups/Descriptives (mean ± SEM, n) | Statistic | p (adjusted) | Effect size [95% CI] | Assumptions |
| --- | --- | --- | --- | --- | --- | --- | --- |
| Cell count | T_CM_ CD38^+^CD69^+^ | Unpaired t-test with Welch's correction | WT veh = 58819 ± 20579 (4); WT LPS = 728003 ± 108227 (6). | t = 6.074, df = 5.356 | WT veh vs. WT LPS p = 0.0014 | 391568 to 946799 | “Non-normal distribution” |
| Cell count | T_EFF/EM_ CD38^+^CD69^+^ | Unpaired t-test with Welch's correction | WT veh = 91971 ± 73897 (4); WT LPS = 432477 ± 87217 (6). | t = 2.979, df = 7.938 | WT veh vs. WT LPS p = 0.0178 | 76539 to 604474 | Shapiro-Wilk (W) p = 0.9592 |

Comparison of T_CM_ and T_EFF/EM_ CD38⁺CD69⁺ cell counts between vehicle- and LPS-treated wild-type mice. Data presented as mean ± SEM; p-values adjusted for multiple comparisons. Unpaired t-tests were applied to homogenize statistical approaches.
